# The Different Response to an Acid Shock of Two *Salmonella* Strains Marks Their Resistance to Thermal Treatments

**DOI:** 10.3389/fmicb.2021.691248

**Published:** 2021-09-20

**Authors:** Marta Clemente-Carazo, José-Juan Leal, Juan-Pablo Huertas, Alberto Garre, Alfredo Palop, Paula M. Periago

**Affiliations:** ^1^Departamento Ingeniería Agronómica, Campus de Excelencia Internacional Regional "Campus Mare Nostrum", Instituto de Biotecnología Vegetal, Escuela Técnica Superior de Ingeniería Agronómica, Universidad Politécnica de Cartagena, Cartagena, Spain; ^2^Food Microbiology, Wageningen University & Research, Wageningen, Netherlands

**Keywords:** foodborne pathogens, acid shock, pasteurization, cross-resistance, stress adaptation

## Abstract

Microbial cells respond to sub-lethal stresses with several physiological changes to increase their chance of survival. These changes are of high relevance when combined treatments (hurdle technology) are applied during food production, as the cells surviving the first hurdle may have greater resistance to subsequent treatments than untreated cells. In this study, we analyzed if *Salmonella* develops increased resistance to thermal treatments after the application of an acid shock. We compared the heat resistance of acid-shocked (pH 4.5 achieved with citric acid) *Salmonella* cells with that of cells maintained at pH 7 (control cells). Thermal treatments were performed between 57.5 and 65°C. We observed a differential response between the two strains studied. Acid-shocked cells of *Salmonella* Senftenberg exhibited reduced heat resistance, e.g., for a treatment at 60.0°C and pH 7.0 the time required to reduce the population by 3 log cycles was lowered from 10.75 to 1.98min with respect to control cells. *Salmonella* Enteritidis showed a different response, with acid-shocked cells having similar resistance than untreated cells (the time required to reduce 3 log cycles at 60.0°C and pH 7.0 was 0.30min for control and 0.31min for acid-shock cells). Based on results by differential plating (with or without adding the maximum non-inhibitory concentration of NaCl to the recovery medium), we hypothesize that the differential response between strains can be associated to sub-lethal damage to the cell membrane of *S*. Senftenberg caused by the acid shock. These results provide evidence that different strains of the same species can respond differently to an acid shock and highlight the relevance of cross-resistances for microbial risk assessment.

## Introduction

Food products can be a potential health hazard for consumers because pathogenic microorganisms may enter the farm-to-fork chain and survive or multiply until consumption (FAO, 2011). Although governmental agencies and other institutions have made intensive efforts toward developing laws and guidelines to ensure food safety, there are still large knowledge gaps in the field. Research efforts are needed to better describe the response of foodborne pathogens to the environmental conditions within the food chain. Among them, *Salmonella* is one of the main safety concerns for the food industry. It causes salmonellosis and is the most frequently detected causative agent of foodborne outbreaks in humans in the European Union (Pires et al., 2019). In 2018, 11,581 outbreak-related human cases of salmonellosis were reported in the European Union; however, this number was lower than the number of total foodborne outbreaks in 2019 (EFSA, 2019, 2021). *Salmonella* Enteritidis was the serovar most commonly isolated in salmonellosis outbreaks (Manas et al., 2001), accounting for 84.1% of them in the European Union in 2018 (EFSA, 2019). This serovar is able to grow in a wide range of NaCl concentrations (between 0 and 4%), acid pH (down to 4.5), water activities (minimum 0.93), and temperatures (between 10 and 45°C; Mattick et al., 2000; Álvarez-Ordóñez et al., 2010; Zurera-Cosano et al., 2011). European Union Regulation (EC) 2073/2005 considers *Salmonella* as a food safety criterium, setting a limit of “not detected in 25g or ml” in five samples, for most food categories.

*Salmonella* can be found in all warm-blooded organisms and the environment, so it can potentially be present in the raw materials used for food production (EFSA, 2019). Hence, one or more steps of food production are usually designed to ensure that the microbial count of this microorganism (and/or other pathogens) is below a threshold concentration. The most common technology for food preservation is the application of high temperatures. Heat treatments are not only effective to ensure microbiological stability and inhibit alterations caused by pathogens, but it can also lead to enzymatic inactivation (Peng et al., 2017). However, high temperatures can also have a negative impact on the quality of the product, reducing its organoleptic and nutritional attributes (Kilcast and Subramaniam, 2011; González-Tejedor et al., 2017; Peng et al., 2017; Maza et al., 2019). For this reason, industries seek a reduction of the intensity of the processing treatments, delivering safe products with a minimal quality loss (minimally processed products).

The application of alternative technologies to substitute or complement thermal treatments was suggested as a strategy to reduce their impact on food quality several decades ago. Leistner (1978) proposed the so-called “hurdle technology,” where mild treatments of a different nature are applied in sequence or simultaneously. According to this approach, food safety can be ensured while reducing the intensity of each individual treatment, improving food quality, and reducing economic cost. On the other hand, due to the combination of several treatments, the hurdle technology requires a deeper knowledge of the process, and a more detailed, science-based understanding of the microbial response to each of the processing treatments (Santos and Silva, 2008). For instance, the application of a treatment that combines high temperature and low pH requires a description of how the microbial cells respond to both the high temperature and the acidic environment.

The principles of the hurdle technology assume that cells that are able to survive the first “hurdle” will be destroyed by the next “hurdles.” However, it is important to take into account that bacterial resistance to stress is affected by the previous history of the cells (Manas and Pagán, 2005; Garre et al., 2019): Sub-lethal damage can alter cellular structures in a reversible or irreversible way, altering their stress resistance (Gilbert, 1984; Hurst, 1984; Yousef and Courtney, 2003). In the particular case of physiological responses to an acid shock, the physiological changes due to stress are denominated acid-shock response (ASR; Zhao et al., 1993; Miller and Kaspar, 1994; Leyer et al., 1995), and several articles have reported that ASR can increase the resistance of microbial cells to subsequent stressors (Kwon and Ricke, 1998; Bang et al., 2002; Rishi et al., 2005). This phenomenon can be of high relevance for food production, as demonstrated by Álvarez-Ordóñez et al. (2012), who observed that, although pathogenic microorganisms were unable to grow in fruit juice, incubation in this medium improved their resistance to subsequent acidic conditions.

Due to the combination of different stresses, food processing based on the hurdle technology can also induce ASR. For instance, it is common for some products to be washed in an acidic solution before they are processed further. Other food products are acidified before being exposed to thermal treatment. Hypothetically, this could induce an ASR in the surviving cells increasing their resistance to a subsequent thermal treatment or other different stresses (Hurst, 1984; Wesche et al., 2009). For that reason, treatments based on the hurdle technology should be designed based on an understanding of not just how the cells respond to each stress, but also to the possible interactions between treatments.

There is plenty of scientific evidence suggesting that the microbial response to stress can vary broadly among different strains of the same microorganism (Koutsoumanis and Aspridou, 2017). For that reason, in this study, we analyzed the heat resistance of acid-shocked cells compared to the one of the cells kept at neutral pH. The study was done by exposing cells to heat after exposure to either pH 4.5 or 7 to elucidate how the ASR may affect the chance of the cells to survive the thermal treatment. Moreover, the experiments were done using two different non-typhoidal *Salmonella* strains: *S*. Enteritidis (a common reference strain) and *S*. Senftenberg (a strain with exceptionally high thermal resistance, according to Álvarez-Ordóñez et al., 2009). This allowed us to also study the potential effect of biological variability, a topic that is currently of high interest for microbial risk assessment (Committee et al., 2018; Clemente-Carazo et al., 2020; Garre et al., 2020).

## Materials and Methods

### Bacterial Culture and Media

The bacteria studied in this research were *Salmonella enterica* serovar Enteritidis CECT 4300 (type strain) and *Salmonella enterica* serovar Senftenberg CECT 4565. Both were provided by the Spanish Type Culture Collection (CECT, Valencia, Spain). *S*. Enteritidis was selected because it is usually considered as a reference strain for this species. *S*. Senftenberg was studied because it is a well-known heat-resistant strain.

The bacteria were stored at −80±2°C (20% glycerol) until use. Subsequently, for the use of each strain, the bacteria were cultured weekly on trypticase soy agar (TSA, Scharlau Chemie, Barcelona, Spain) supplemented with 0.6% yeast extract (YE, Scharlau Chemie). The fresh cultures were incubated for 24h at 37±1°C in an incubator.

A single colony from a fresh culture plate was transferred to 5ml of trypticase soy broth (TSB; Scharlau Chemie) supplemented with 0.6% yeast extract (YE, Scharlau Chemie) and incubated overnight at 37±1°C. Flasks with 50ml of TSBYE were then inoculated with 1ml of the pre-culture and incubated for 24h at 37±1°C with agitation to obtain all cells in stationary phase, which usually are more resistant to different stresses, with a concentration of approx. 10^9^CFU/ml.

### Determination of Minimum pH for Growth

Sterile TSB (Scharlau Chemie) supplemented with 0.6% yeast extract (YE, Scharlau Chemie) was prepared at different pH (3.75, 4.00, 4.25, 4.50, and 7.00) by the addition of 1 M citric acid (Panreac, Barcelona, Spain). The pH was measured after sterilization with a pH meter (Basic20, Crison; Alella, Spain), under strict aseptic conditions, to check there were no changes in the pH. Citric acid was chosen to lower the pH of the growth medium as it is one of the most common acidulants used to lower the pH in the food industry.

Then, the growth of both Salmonella serotypes in the acidified TSBYE was determined in a Bioscreen C (Labsystems Helsinki, Finland) at a wavelength of 600nm. A total of 25 repetitions per pH value were performed, of which five were left uninoculated and used as negative control. Samples at pH 7.00 were used as positive control.

### Induction of the Acid Tolerance Response (Acid Shock)

A volume of 1ml of cells in the stationary growth phase (10^9^CFU/ml) of each serotype was centrifuged at 6,000g (MiniSpin^®^ plus, Eppendorf AG, Germany) for 10min at 4±1°C. Pellets were resuspended in pH 7.0 TSBYE and centrifuged two more times and resuspended in fresh medium. After the last centrifugation, pellets were resuspended in pH 4.5 TSBYE and were incubated at 37±1°C for 30min in an incubator and immediately heat treated.

### Determination of the Heat Resistance

The determination of the heat resistance of the microorganisms was carried out in a Mastia thermoresistometer (Conesa et al., 2009). The heating medium for every experiment was peptone water [10g/l peptone from casein (Scharlau Chemie) and 5g/l NaCl (Scharlau Chemie)]. For the experiments that were conducted in acidic media, peptone water was acidified with 1 M citric acid to pH 4.5 and measured with a pH meter.

The heating medium was inoculated with 0.2ml of the bacterial suspension (acid shocked and control) with a concentration of approximately 10^6^CFU/ml. Experiments were performed under isothermal condition. The temperatures and sampling times were set according to the thermal resistance of each microorganism and conditions. For Salmonella Enteritidis, experiments were performed at 52.5, 57.5, and 60.0°C at both pH 7.0 and pH 4.5. For *Salmonella* Senftenberg, experiments were carried out at higher temperatures (57.5, 60.0, 62.5, and 65.0°C) at both pH values (7.0 and 4.5) because of the higher thermal resistance of this strain.

Survival counting was performed, from appropriate dilutions in peptone water, in TSAYE or TSAYE + NaCl (for the determination of sub-lethal damage; see section “Assessment of Sub-Lethal Damage After Treatments”). A minimum of three biologically independent replicate experiments was performed per condition.

### Assessment of Sub-Lethal Damage After Treatments

The concentration of cells with sub-lethal damage after the acid shock was estimated by differential plating in TSAYE and TSAYE supplemented with the maximum non-inhibitory concentration of NaCl and incubation at 37±1°C for 48h (Ray, 1989; Wuytack et al., 2003). The maximum non-inhibitory concentration of NaCl was determined preparing TSAYE with different percentages of NaCl, from 1 to 5%. Then, cells of both strains of *Salmonella* spp. were plated on Petri plates and incubated at 37±1°C for 48h. This concentration resulted in 1% NaCl for *S*. Enteritidis and 3% for *S*. Senftenberg.

### Data Analysis

The isothermal inactivation data were analyzed using the Mafart inactivation model (Mafart et al., 2002). This model extends the log-linear inactivation model assuming that the resistance of individual cells is not the same for all them but follows a Weibull distribution. It thus can describe survivor curves with upward or downward curvature. According to Equation (1), in this model, the relationship between the microbial count (N) and the treatment time (t) depends on two parameters: δ and β. The former (also called δ-value) corresponds to the scale parameter of the Weibull distribution and can be interpreted as the treatment time required to reduce the microbial count to a 10% of the initial one, N_0_. The latter (also called β-value) is the shape factor of the Weibull distribution and defines the curvature direction of the survivor curves: β >1 results in survivor curves with downward curvature and β <1 in upward curvature. For the particular case where β =1, the Mafart model predicts log-linear inactivation. The primary inactivation model was fitted using the Excel add-in GinaFit (Geeraerd et al., 2005).

$$logN=\log N_{0}-{\left(\frac{t}{\delta }\right)}^{\beta }$$. (1)

Model fitting was done using the functions included in the *bioinactivation* R package (Garre et al., 2018), using a one-step approach based on non-linear regression with the Newton–Raphson algorithm. Initial guesses for the parameter estimates were defined based on preliminary simulations, and several values were tested without observing any relevant impact on parameter estimates. The evaluation of the model fit was based on the root mean squared error, a statistical index commonly used in predictive microbiology that quantifies the overall difference between the model fit and the observations, with values closer to one indicating a better correspondence.

Because the β-value of the survivor curves varied between conditions, in order to compare between them, we calculated the treatment time required to reduce the microbial count 3 log cycles according to the Weibull model (t_3δ_). It was calculated as shown in Equation (2).

$$t_{n\delta }=\delta \cdot n^{1/\beta }$$. (2)

## Results

**Figure 1:**
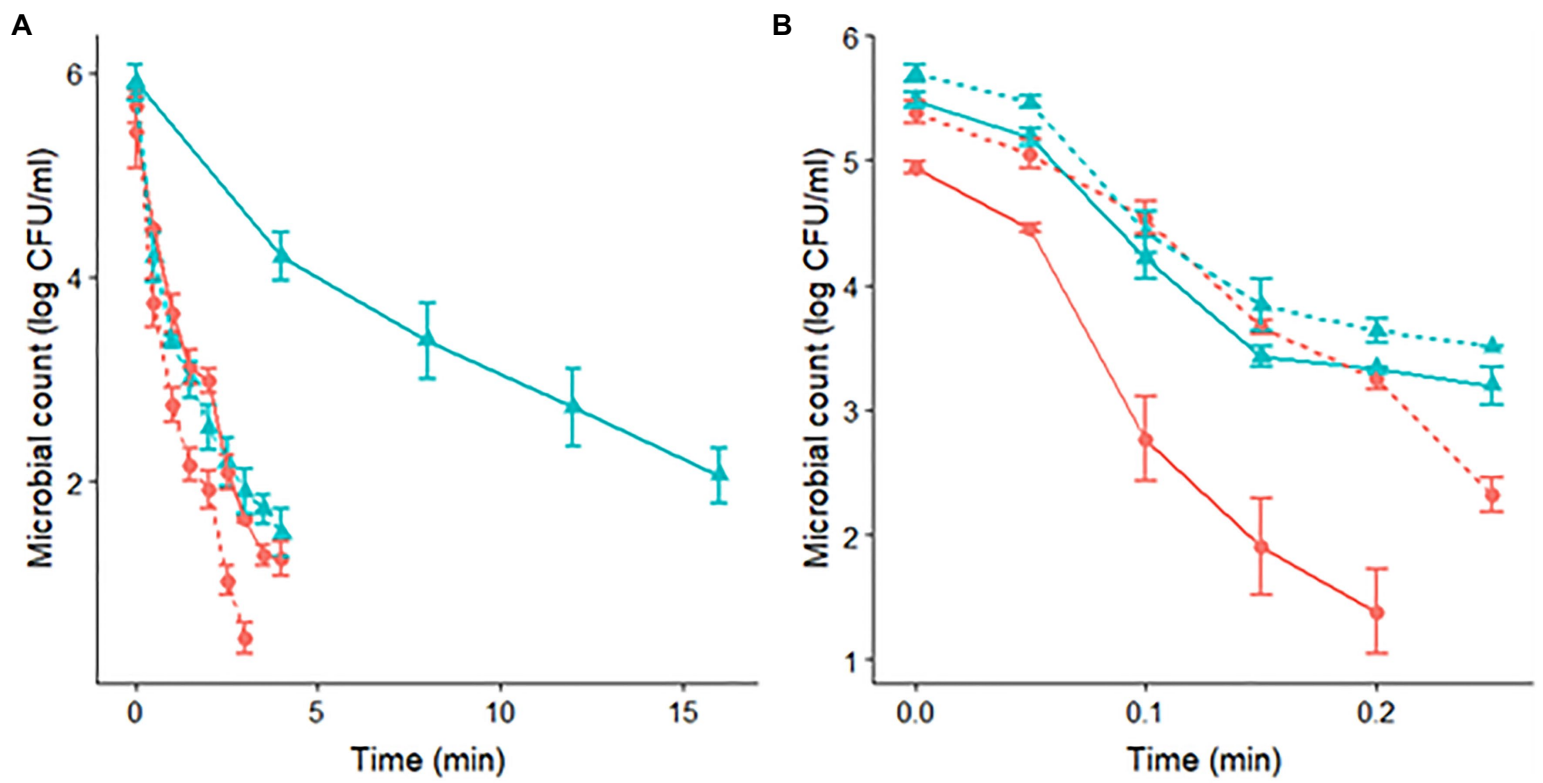
Survivor curves of *S*. Senftenberg **(A)** and *S*. Enteritidis **(B)** during isothermal treatments at 60°C in peptone water. The pH of the heating media was set at pH 7.0 (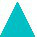) and pH 4.5 (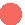). Experiments were made for cells with a previous acid shock (dashed lines) and control cells, without acid shock (continuous lines).

Tables 1 and 2 report the parameters of the Weibull model estimated for each isothermal treatment. They confirm the high resistance of *S*. Senftenberg to thermal treatments compared to the reference strain. As an illustration, for the experiments at 60.0°C at pH 7.0 without a prior acid shock, a value of 10.75min was estimated for t_3δ_ for *S*. Senftenberg, whereas a value of 0.30min was estimated for *S*. Enteritidis (36 times smaller). This difference in the resistance between both strains is also illustrated in Figure 1, where the survivor curves observed at 60.0°C for both microorganisms are depicted. The remaining survivor curves are provided as supplementary material to this article. The inactivation rates are in the order of similar studies found in the literature. Doyle and Mazzotta (2000) reviewed several studies observing that the D-value of *S*. Senftenberg at 60°C in laboratory media ranged between 0.62 and 3.06min. The results are also similar to those by Shah et al. (1991), who observed D-values of *Salmonella* Enteritidis between 9.98min at 54.4°C and 0.05min at 62.8°C.

**Table 1:** Parameters of the Weibull model estimated from the survival curves of *S*. Senftenberg heat treated in peptone water without a prior acid shock (control cells) and after an acid shock at pH 4.5.

	pH	Temperature (°C)	*δ* (min)	*β* (·)	*t_3δ_* (min)	RMSE (log CFU/ml)
Control cells	4.5	57.5	1.53±0.55	0.66±0.12	8.08	0.31
		60.0	0.61±0.12	0.77±0.08	2.54	0.27
		62.5	0.04±0.01	0.46±0.04	0.44	0.29
		65.0	0.11±0.03	1.01±0.16	0.33	0.45
	7.0	57.5	6.61±2.61	0.70±0.15	31.75	0.32
		60.0	1.67±0.51	0.59±0.08	10.75	0.27
		62.5	0.37±0.08	0.51±0.04	3.19	0.17
		65.0	0.16±0.05	0.70±0.11	0.77	0.33
Acid-shocked	4.5	57.5	0.65±0.15	0.55±0.04	4.79	0.21
cells		60.0	0.33±0.09	0.69±0.08	1.62	0.31
		62.5	0.01±0.01	0.30±0.04	0.39	0.31
		65.0	0.05±0.01	0.73±0.09	0.23	0.23
	7.0	57.5	1.42±0.29	0.60±0.06	8.86	0.17
		60.0	0.24±0.05	0.52±0.04	1.98	0.20
		62.5	0.08±0.03	0.47±0.05	0.83	0.26
		65.0	0.0016±0.0013	0.24±0.04	0.16	0.16

**Table 2:** Parameters of the Weibull model estimated from the survival curves of *S*. Enteritidis heat treated in peptone water without a prior acid shock (control cells) and after an acid shock at pH 4.5.

	pH	Temperature (°C)	*δ* (min)	*β* (·)	*t_3δ_* (min)	RMSE (log CFU/ml)
Control cells	4.5	52.5	2.44±0.43	0.72±0.05	11.22	0.26
		57.5	0.14±0.03	0.76±0.08	0.59	0.24
		60.0	0.05±0.01	0.98±0.18	0.15	0.38
	7.0	52.5	6.32±1.84	0.65±0.09	34.26	0.28
		57.5	0.16±0.04	0.62±0.07	0.94	0.28
		60.0	0.08±0.02	0.83±0.16	0.30	0.29
Acid-shocked	4.5	52.5	5.82±2.12	0.76±0.14	24.70	0.43
cells		57.5	0.36±0.04	0.98±0.07	1.10	0.19
		60.0	0.11±0.01	1.34±0.10	0.25	0.13
	7.0	52.5	18.9±0.6	2.15±0.12	31.52	0.18
		57.5	0.34±0.05	1.24±0.18	0.82	0.27
		60.0	0.08±0.02	0.82±0.15	0.31	0.27

Regarding the curvature direction of the inactivation curves, they differed between strains. *S*. Senftenberg (Figure 1A and Table 1) presented a tail phenomenon (upward concavity) in all the treatments applied, reflected in β-values lower than one (for a temperature of 60.0°C: β=0.69±0.08 for pH 4.5 after acid shock, β=0.52±0.04 for pH 7.0 after acid shock, β=0.77±0.08 for pH 4.5 without acid shock, and β=0.59±0.08 for pH 7.0 without acid shock). On the other hand, *S*. Enteritidis showed β-values close to one (Figure 1B and Table 2), thus having survivor curves close to log linear (for a temperature of 60.0°C: β=1.34±0.10 for pH 4.5 after acid shock, β=0.82±0.15 for pH 7.0 after acid shock, β=0.98±0.18 for pH 4.5 without acid shock, and β=0.83±0.16 for pH 7.0 without acid shock).

As expected, the combination of an acidic medium and a heat treatment had a significant impact on the inactivation rate of both strains. As shown in Table 1, *S*. Senftenberg showed a time for 3 log-reductions at pH 4.5 between two and seven times shorter than at pH 7.0 (t_3δ_values at pH 4.5 of 8.08, 2.54, 0.44, and 0.33min at 57.5, 60.0, 62.5, and 65.0°C, respectively, compared to t_3δ_ values at pH 7.0 of 31.75, 10.75, 3.19, and 0.77min at 57.5, 60.0, 62.5, and 65.0°C, respectively). *S*. Enteritidis was less sensitive to thermal treatment under acidic pH (Table 2), showing a time to reach 3 log-reductions at pH 4.5 between only two and three times shorter than at pH 7.0 (t_3δ_ values at pH 4.5 of 11.22, 0.59, and 0.15min at 52.5, 57.5, and 60.0°C, respectively, compared to t_3δ_ values at pH 7.0 of 34.26, 0.94, and 0.30min at 52.5, 57.5, and 60.0°C, respectively; Table 2).

The application of an acid shock prior to the thermal treatment had a different impact on the thermal resistance of both strains. For *S*. Senftenberg, the survivor curves at pH 7.0 after acid shock showed a significantly faster inactivation rate than the one of control cells. The application of the acid shock reduced t_3δ_at 60°C from 10.75min for control cells to 1.98min for acid-shocked cells. Notably, the value of t_3δ_observed for acid-shocked cells at pH 7.0 (1.98min) was about the same as that observed at pH 4.5 without any previous acid shock (2.54min). This similarity between inactivation under both conditions is clearly illustrated in Figure 1A. Therefore, the application of an acid shock prior to a treatment at pH 7 had a similar impact on the inactivation kinetics than a combined thermal treatment at pH 4.5. Regarding the effect of an acid shock before a thermal treatment at pH 4.5 for *S*. Senftenberg, although the stress resistance was reduced, the magnitude of this reduction was not as large as for the treatments at pH 7. The value of t_3δ_ was reduced from 2.54 to 1.62min at 60°C. Similar results were obtained at all other temperatures tested (Table 1). In summary, the application of an acid shock to *S*. Senftenberg reduced its thermal resistance. Indeed, the inactivation kinetics of acid-shocked cells at a pH of 7.0 was similar to the one of control cells at pH 4.5. For treatments with a pH of 4.5, the thermal resistance of acid-shocked cells was further reduced.

Conversely, *S*. Enteritidis had a very different response to the application of an acid shock. As shown in Table 2, acid-shocked cells of *S*. Enteritidis had a resistance to the thermal treatment in both acidic and neutral media equivalent to the one observed for control cells at pH 7 (t_3δ_ at 60.0°C of 0.30min for control cells at pH 7.0, 0.31min for acid-shocked cells treated at pH 7.0, and 0.25min for acid-shocked cells treated at pH 4.5). These similarities are illustrated in Figure 1B, where the survivor curves obtained at 60.0°C are illustrated. This plot shows how the survivor curves for acid-shocked cells for a treatment pH of 4.5 and 7.0 are about the same as the one observed for control cells for a treatment pH of 7.0. This result can indicate that the acid shock causes an ASR that induces an increase in the thermal resistance of *S*. Enteritidis cells, making them insensitive to changes in the acidity of the heating medium in subsequent treatments.

**Figure 2:**
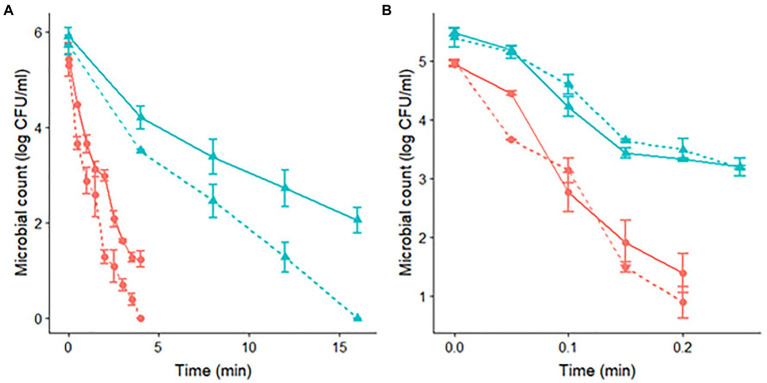
Survivor curves of *S*. Senftenberg **(A)** and *S*. Enteritidis **(B)** during isothermal treatments at 60°C in peptone water and recovered in TSAYE (continuous lines) and TSAYE with NaCl (dashed lines) for experiments performed at pH 7.0 (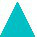) and pH 4.5 (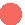).

The data obtained using differential plating with NaCl provide further evidence to support the hypothesis that ASR is responsible for the different results observed between the two strains. Figure 2 compares the microbial counts observed in TSAYE and TSAYE supplemented with NaCl. For *S*. Senftenberg, we observed microbial counts between 1 and 2 log CFU/mL lower in TSAYE + NaCl than in TSAYE at both pH levels (7.0 and 4.5). This is an indicator of sub-lethal damage in the microbial cells that could justify our results, where acid-shocked cells had a lower heat resistance than control cells (Figure 2A). On the other hand, we did not observe any difference between the microbial counts recovered in TSAYE and TSAYE + NaCl for *S*. Enteritidis, indicating the absence of sub-lethal damage for this strain. This could enable the development of ASR of the cells that could induce an increased thermal resistance, justifying our results (Figure 2B).

## Discussion

The addition of chemicals (e.g., salts, acids, and oxidizers) or the application of physical treatments (e.g., heat and pressures) for food preservation induce a variety of environmental stresses that can inactivate pathogenic and spoilage microorganisms present in the food product. However, current processing treatments are not flawless, evidenced by the fact that a number of foods with an acidic pH have been implicated in outbreaks. Indeed, some studies have questioned the effect of acid on the effective control of some microorganisms (Parish, 1997; Evans et al., 1999; Rodriguez-Romo et al., 2005; Vojdani et al., 2008; Jain et al., 2009). Furthermore, previous studies have demonstrated that acidic conditions may improve the resistance of some strains of *Salmonella* spp. An example is the work by Perez et al. (2010), who showed that incubation in acidic condition led to an increase in the survival of *S*. Enteritidis in gastrointestinal liquids, due to prior adaptation to an acidic pH. The ability of *Salmonella spp*. to survive this type of stresses and their presence in a wide variety of foods makes it a high-risk pathogen (Rychlik and Barrow, 2005). As a result, an accurate risk assessment for this microorganism requires to understand the physiological state of the cells and the potential for a stress adaptation (Álvarez-Ordóñez et al., 2013). This is especially relevant as several strains of *Salmonella* can develop an increased stress resistance when exposed to acidic conditions, including *S*. Enteritidis and *S*. Senftenberg (Hsin-Yi and Chou, 2001; Greenacre et al., 2003; Koutsoumanis et al., 2003; Koutsoumanis and Sofos, 2004; Stopforth et al., 2007; Skandamis et al., 2009; Álvarez-Ordóñez et al., 2012).

In the current study, we have observed a differential response in two *Salmonella* strains when exposed to an acid shock. The acid-shocked *S*. Senftenberg cells became more sensitive to the thermal treatment, whereas *S*. Enteritidis showed the opposite response. For treatments at pH 7, the resistance of acid-shocked cells was practically the same as the one of control cells. Moreover, acid-shocked cells of *S*. Enteritidis were practically insensitive to changes in the pH of the heating medium in our experiments (practically the same t_3δ_ values at pH 7 and 4.5). These results are in-line with those by Malheiros et al. (2009), who observed that at temperatures of 52.0 and 56.0°C *S*. Enteritidis was able to survive, showing higher adaptation capabilities than other serovars of this species in a heating medium in the presence of glucose at pH 4.5. A similar physiological response (albeit for a different microorganism) was reported by Haberbeck et al. (2017). In their study, they observed that 48 *Escherichia coli* strains were able to adapt after incubation at pH 5.5, increasing their thermal resistance to treatments at 58.0°C. Not all the studies confirm these results, since other authors showed an increased sensitivity to heat when exposed to low pH in this same pathogen of *E. coli* (Lee and Kang, 2009; Parry-Hanson et al., 2010). Our empirical results demonstrate that very different responses can be observed in the ability of different strains of a microorganism to develop an increased stress resistance after being subjected to an acid shock.

Conversely to *S*. Enteritidis, we observed that for *S*. Senftenberg the application of an acid shock increased the sensitivity of the bacteria to posterior thermal treatments. Based on our observations using differential plating, this result could be attributed to sub-lethal damage in the cytoplasmic membrane of *S*. Senftenberg cells. This result is in disagreement with those reported by Álvarez-Ordóñez et al. (2009), who observed that acid-adapted cells of *S*. Senftenberg would develop increased stress resistance. However, these authors allowed the growth of the cells in acidified media for a long time, while we applied a short acid shock. They also used food matrices as heating media (orange juice and apple juice), whereas in our study we used laboratory media. There is empirical evidence showing that food matrices can have a protective effect on microbial cells (Maté et al., 2017; Verheyen et al., 2019, 2020). Hence, it is a plausible hypothesis that the food matrix shall also influence the development of increased stress resistance.

During the last decades, the application of modern technologies (e.g., genomics) have shed light on the genes involved in the microbial response to stress, and the molecular mechanisms that intercede in the cellular response to acidic conditions. Due to low pH environments, protons are expelled from the cytoplasm to regulate pH levels within the cell. This is carried out with potassium-proton and sodium-proton antiporter pumps, keeping the intracellular pH constant (Foster, 2000). Previous studies have also shown that through the synthesis of acid-shock proteins and regulatory proteins, the cell is able to avoid or repair the damage caused by the acid stress. These molecular mechanisms can explain the empirical evidence supporting the hypothesis that microbial cells are able to recover from the damage caused by an acid stress (Audia et al., 2001).

## Conclusion

Our results demonstrate that the response to an acid shock of *Salmonella* cells depends very much on the strains of this species. The application of an acid shock strongly reduced the heat resistance of *S*. Senftenberg, whereas the same shock induced a stress response in *S*. Enteritidis, increasing its heat resistance. These responses may probably be representative of extreme behaviors within strains of this species and should be taken into account when applying an acid wash or even acidifying food products prior to exposing them to a thermal preservation treatment. This result can also help advance risk assessment, reducing the uncertainty associated to the microbial response in acidic food products.

## Data Availability Statement

The original contributions presented in the study are included in the article/Supplementary Material, further inquiries can be directed to the corresponding author.

## Author Contributions

MC-C, J-JL, J-PH, AG, AP, and PP: conceptualization. MC-C and AG: formal analysis. MC-C and JJL: investigation. MC-C: writing—original draft preparation. MC-C, AG, AP, and PP: writing—review and editing. J-PH, AG, AP, and PP: supervision. All authors have read and agreed to the published version of the manuscript.

## Funding

This research was funded by the Ministry of Science, Innovation and Universities of The Spanish Government and European Regional Development Fund (ERDF), grant numbers AGL2017-86840-C2-1-R and PID2020-116318RB-C32. Alberto Garre was supported by a postdoctoral grant from the Fundacion Séneca grant number 20900/PD/18 and by the European Union’s Horizon 2020 Research and Innovation Programme under the Marie Sklodowska-Curie grant number 844423 (FANTASTICAL).

## Conflict of Interest

The authors declare that the research was conducted in the absence of any commercial or financial relationships that could be construed as a potential conflict of interest.

## Publisher’s Note

All claims expressed in this article are solely those of the authors and do not necessarily represent those of their affiliated organizations, or those of the publisher, the editors and the reviewers. Any product that may be evaluated in this article, or claim that may be made by its manufacturer, is not guaranteed or endorsed by the publisher.
