# Supplementary material for: The Different Response to an Acid Shock of Two Salmonella Strains Marks Their Resistance to Thermal Treatments
Source: Front Microbiol. 2021 Sep 20;12:691248. doi: 10.3389/fmicb.2021.691248 (PMC8488367; doi:10.3389/fmicb.2021.691248)
Supplement: Supplementary file 1 [file Data_Sheet_1.docx]

Supplementary Material


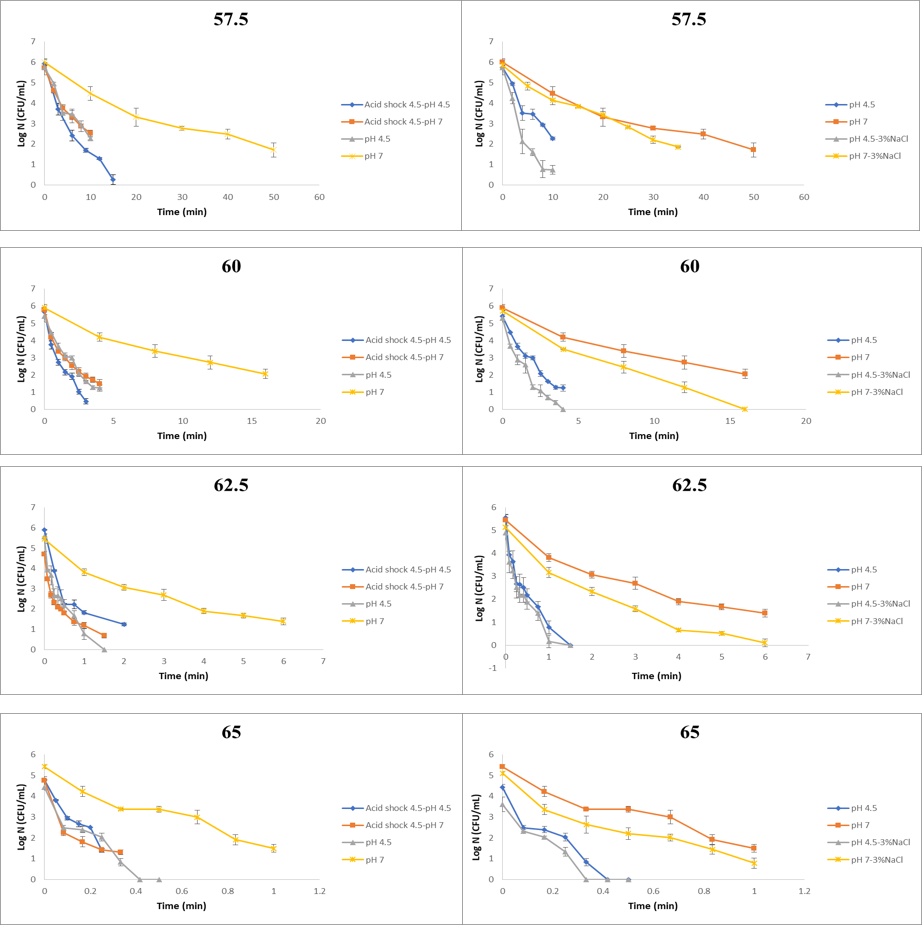


Figure S1. Inactivation kinetics at different temperatures and determination of sublethal damage with TSA+NaCl for Salmonella Senftenberg.


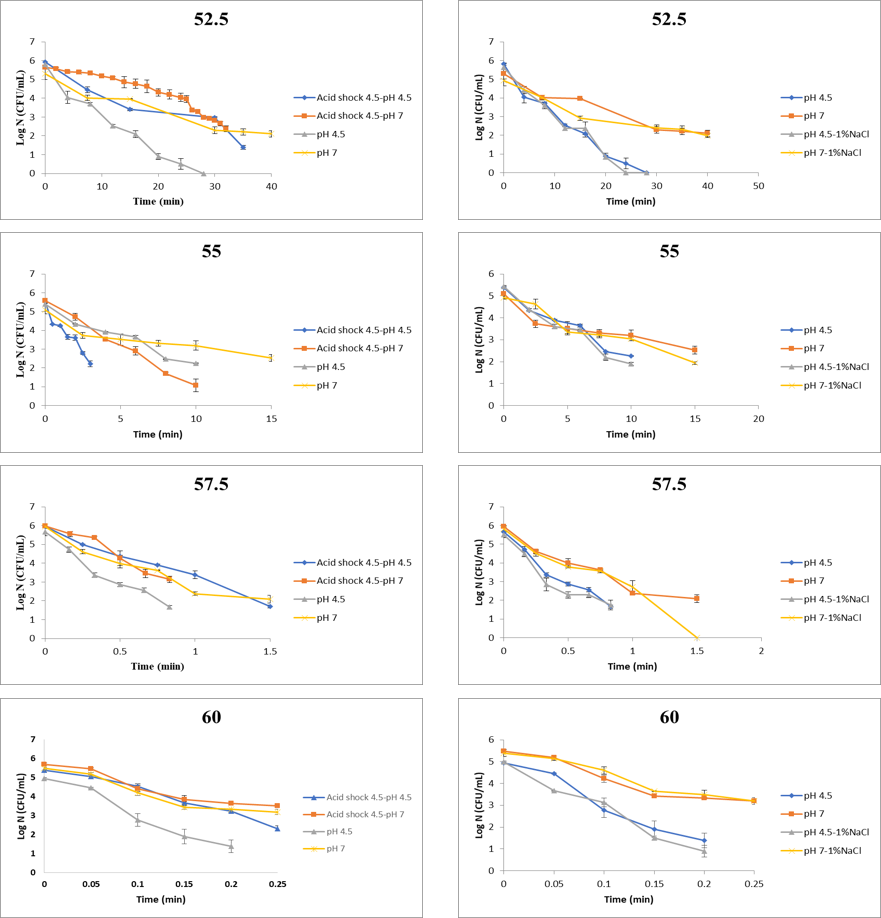


Figure S2. Inactivation kinetics at different temperatures and determination of sublethal damage with TSA+NaCl for Salmonella Enteritidis.

Figure S3. Determination of the minimum growth pH for both serovars.
